# PrtA immunization fails to protect against pulmonary and invasive infection by *Streptococcus pneumoniae*

**DOI:** 10.1186/s12931-018-0895-8

**Published:** 2018-09-25

**Authors:** Chen-Fang Hsu, Chen-Hao Hsiao, Shun-Fu Tseng, Jian-Ru Chen, Yu-Jou Liao, Sy-Jou Chen, Chin-Sheng Lin, Huey-Kang Sytwu, Yi-Ping Chuang

**Affiliations:** 1 Department of Pediatrics, Chi Mei Medical Center, Tainan, Taiwan; 2 Taipei Medical University, Taipei, Taiwan; 3 Kaohsiung Medical University, Kaohsiung, Taiwan; 4 Chung Shan Medical University, Taichung, Taiwan; 5 Cheng Hsin General Hospital, Taipei, Taiwan; 6 Genome and Systems Biology Degree Program, National Taiwan University and Academia Sinica, Taipei, Taiwan; 7 Department and Graduate Institute of Microbiology and Immunology, National Defense Medical Center, Taipei, Taiwan; 8 Department of Emergency Medicine, Tri-Service General Hospital, National Defense Medical Center, Taipei, Taiwan; 9 Graduate Institute of Injury Prevention and Control, College of Public Health and Nutrition, Taipei Medical University, Taipei, Taiwan; 10 Division of Cardiology, Department of Medicine, Tri-Service General Hospital, National Defense Medical Center, Taipei, Taiwan; 11 National Institute of Infectious Diseases and Vaccinology, National Health Research Institutes, Miaoli, Taiwan

**Keywords:** *Streptococcus pneumoniae*, IL-17A, PrtA, Curdlan

## Abstract

**Background:**

*Streptococcus pneumoniae* is a respiratory pathogen causing severe lung infection that may lead to complications such as bacteremia. Current polysaccharide vaccines have limited serotype coverage and therefore cannot provide maximal and long-term protection. Global efforts are being made to develop a conserved protein vaccine candidate. PrtA, a pneumococcal surface protein, was identified by screening a pneumococcal genomic expression library using convalescent patient serum. The *prtA* gene is prevalent and conserved among *S. pneumoniae* strains. Its protective efficacy, however, has not been described. Mucosal immunization could sensitize both local and systemic immunity, which would be an ideal scenario for preventing *S. pneumoniae* infection.

**Methods:**

We immunized BALB/c mice intranasally with a combination of a PrtA fragment (amino acids 144–1041) and Th17 potentiated adjuvant, curdlan. We then measured the T-cell and antibody responses. The protective efficacy conferred to the immunized mice was further evaluated using a murine model of acute pneumococcal pneumonia and pneumococcal bacteremia.

**Results:**

There was a profound antigen-specific IL-17A and IFN-γ response in PrtA-immunized mice compared with that of adjuvant control group. Even though PrtA-specific IgG and IgA titer in sera was elevated in immunized mice, only a moderate IgA response was observed in the bronchoalveolar lavage fluid. The PrtA-immunized antisera facilitated the activated murine macrophage, RAW264.7, to opsonophagocytose *S. pneumoniae* D39 strain; however, PrtA-specific immunoglobulins bound to pneumococcal surfaces with a limited potency. Finally, PrtA-induced immune reactions failed to protect mice against *S. pneumoniae-*induced acute pneumonia and bacterial propagation through the blood.

**Conclusions:**

Immunization with recombinant PrtA combined with curdlan produced antigen-specific antibodies and elicited IL-17A response. However, it failed to protect the mice against *S. pneumoniae*-induced infection.

**Electronic supplementary material:**

The online version of this article (10.1186/s12931-018-0895-8) contains supplementary material, which is available to authorized users.

## Background

*Streptococcus pneumoniae* is a gram-positive encapsulated coccus that generally colonizes the upper respiratory tract in humans without symptoms [1]. However, it may cause community-acquired pneumonia and invasive infections owing to mucosal translocation, such as bacterial meningitis, bacteremia, and otitis media [2, 3]. Therefore, *S. pneumoniae* remains the most common acute pneumonia-causing pathogen in infants and the elderly worldwide [4, 5]. Not only is it a common cause of primary bacterial pneumonia, *S. pneumoniae* also frequently causes secondary bacterial pneumonia following influenza virus infection, thus becoming the main cause of high mortality in adults [6, 7].

Considering *S. pneumoniae*’s impact on morbidity and mortality, healthcare providers promote vaccination to prevent pneumococcal infection. PPSV23 and conjugate vaccines PCV10 and PCV13 are currently available for use as vaccines [8, 9]. The unconjugated polysaccharide PPSV23 vaccine provides higher coverage of serotypes against pneumococci than the other vaccines, but it cannot be administered to infants owing to their underdeveloped immune system. The conjugated vaccine has reduced the occurrence of invasive pneumococcal diseases since PCV7 was introduced in 2000 [10, 11]; however, cases of pneumococcal infections increased because of non-vaccine serotypes [12, 13]. In fact, invasive diseases attributable to serotypes that are included in the current polysaccharide vaccines may still threaten the protective effect [14]. Notably, capsular polysaccharide vaccines are less effective against acute otitis media and non-bacteremic pneumococcal pneumonia in adult populations [15, 16], possibly due to capsule shedding in response to the epithelium during mucosal infection [17]. Therefore, efforts are being made to develop vaccines containing highly conserved, immunogenic protein antigens, which are selected by immunoscreening with the patients’ sera [18] or by reverse vaccinology [19], among other approaches.

PrtA, a cell wall-associated protein, was screened from convalescent patient serum after *S. pneumoniae* infection [20] and was identified as a serine protease. The amino terminal of PrtA, containing catalytic domains, was highly conserved among 78 clinical pneumococcal isolates displaying 22 different serotypes, including the D39 strain [21]. The deletion of the *prtA* in *S. pneumoniae* D39 reduced mortality at 36 h after intraperitoneal infection [21] and alleviated lung inflammation at 48 h after intranasal infection [22]. Furthermore, *prtA* expression in *S. pneumoniae* could be induced during epithelial cell contact, pneumococcal bacteremia, and meningitis in infected mice [23]. Although the immunogenicity, conservation, and virulence of PrtA have been reported, the efficacy of PrtA as a vaccine against pneumococcal infection has not been studied.

The Th17 response is considered effective against *S. pneumoniae* infection. The intratracheal administration of recombinant IL-17A can stimulate the local release of MIP-2 and IL-1β, leading to the recruitment of polymorphonuclear leukocytes to the lungs [24]. Th17 response also triggers the mucosal epithelium to generate anti-microbial peptides, which facilitate the elimination of mucosal pathogens [25]. Mice lacking the IL-17A receptor, but not IFN-γ or IL-4 receptors, demonstrated decreased protection against *S. pneumoniae* [26]. Thus, screening for Th17-based antigens is a feasible approach to select vaccine candidates that could reduce *S. pneumoniae* colonization [27]. Adjuvants provide an alternative approach to support ideal vaccine candidates and develop appropriate cellular immunity.

Curdlan is a linear and nonionic β-1,3-glucan isolated from the bacterium *Alcaligenes faecalis*. Due to its non-toxicity [28, 29] and heat-based gel-forming capabilities [30], curdlan has been approved as a food additive by the U.S. Food and Drug Administration. Curdlan is an agonist of Dectin-1, activating dendritic cells to induce Th17 differentiation [31], along with being a strong inducer of Th17 response [32]. Therefore, curdlan has been successfully adopted as a vaccine adjuvant against *Pseudomonas aeruginosa* pulmonary infection [33], and lethal Candida infection [34], among other infections [35]. In the present study, we investigated the efficacy of PrtA immunization combined with curdlan to prevent *S. pneumoniae* infection. We used BALB/c mice to deliver the vaccine via the nasal route to trigger both local mucosal and systemic immune responses, we also checked the vaccines’ capability to reduce the severity of pneumonia as well as the incidence of invasion.

## Methods

### Bacterial strains and media

*Escherichia coli* strains DH5α and BL21 (DE3) were used for plasmid cloning and protein expression, respectively. The strains harboring plasmids were grown in Luria–Bertani medium supplemented with kanamycin (50 μg/ml). *S. pneumoniae* D39 (NCTC7466) was purchased from the National Collection of Type Cultures (London, UK), and *S. pneumoniae* TIGR4 (ATCC BAA-334) was obtained from American Type Culture Collection (Manassas, VA, USA). *S. pneumoniae* strains were cultured at 37 °C in 5% CO_2_ in Todd–Hewitt broth supplemented with 0.5% yeast extract (THY) until mid-log phase for the bacterial challenge, and on sheep-blood agar plates for bacterial load examination after infection.

### Construction of expression vector

The gene encoding a PrtA fragment (amino acids 144–1041) was cloned into plasmid pET29a using the primer pair F1-AATCGAGCTCCTATCCAATC and R1-TGAGCCTCGAGAGGATTTCC to construct pET-PrtA105-His. The underlined primer sequences were modified to obtain appropriate restriction sites. To generate recombinant PrtA with dual tags, another primer pair, F2-AGGGTACCGTATTCATGTCC and R2-GCAGATCGTCAGTCAGTCAC, was used to clone DNA encoding glutathione S transferase (GST) from pGEX-4 T-1, which was inserted into pET-PrtA105-His to construct pET-GST-PrtA105-His.

### Purification of recombinant proteins

*E. coli* BL21 (DE3) was transformed with an expression vector. Protein expression was induced in the exponential phase [optical density (OD) at 600 nm = 1] using 1 mM isopropyl β-D-1-thiogalactopyranoside (IPTG). At 3 h after induction, the bacteria were harvested and resuspended in lysis buffer [20 mM Tris (pH 8.0), 5 mM imidazole, 500 mM NaCl, 10% glycerol, 1% Triton X-100, 1 mM phenylmethylsulfonyl fluoride, 1× protease inhibitor cocktail (Roche)], followed by sonication on ice. The recombinant protein PrtA105-His (PrtA1) was purified using Ni-NTA resin (GE Healthcare) according to the manufacturer’s instructions. The other recombinant protein, GST-PrtA105-His (PrtA2), was further purified with glutathione-Sepharose® 4B (Millipore). Lipopolysaccharide (LPS) contamination was reduced by using 0.1% Triton X-114 in the washing step [36]. The residual LPS was less than 1 EU/μg protein, as determined by a ToxinSensor™ Gel Clot Endotoxin Assay Kit (GenScript).

### Mice

BALB/c mice used in the immunization and challenge experiments were purchased from the National Laboratory Animal Center, NARLabs, Taiwan.

### Mice immunization

Male mice (3–4 weeks old) were treated intranasally with 8 μg recombinant PrtA1 combined with 0.2 mg curdlan (Sigma) in 20 μl phosphate-buffered saline (PBS) once a week for 3 weeks under anesthesia (intramuscular injection of 40 μl PBS containing Zoletil 50 and 1% Rompun®). One week after the last immunization, mice were restrained to collect saliva before sacrificing them to collect nasal washes, sera, bronchoalveolar lavage fluid (BALF), and spleens.

For PPSV23 vaccination, Pneumovax®23 (Merck & Co., Inc.) vaccines were 10^− 1^ diluted in saline, and 100 μl was injected intraperitoneally into five-week-old mice. Booster immunizations were administered after 2 weeks.

### Identification of CD4^+^ T cell subsets

CD4^+^ T cell subsets (Th1 [IFN-γ-producing CD4^+^ T cells], Th2 [IL-4-producing CD4^+^ T cells], and Th17 [IL-17A-producing CD4^+^ T cells]) were identified by intracellular cytokine staining. To estimate the total effector T cell ratio, splenocytes (2 × 10^6^ cell/ml) were seeded in 24-well plates and stimulated with phorbol 12-myristate13-acetate (PMA) (20 ng/ml) and ionomycin (1 μM) for 6 h while incubating with 4 μM monensin. The antibodies used were FITC-anti-IFN-γ (XMG1.2), PE-anti-IL-4 (11B11), APC-anti-CD4 (RM4–5) (eBioscience), and PE-anti-IL-17A (TC11-18H10) (BD Biosciences). Intracellular staining was performed after treatment with permeabilization buffer (eBioscience) containing 0.1% saponin according to the instructions provided by eBioscience, and the effector T cell ratio was analyzed by flow cytometry using a FACSCalibur™ (BD Biosciences).

### PrtA-specific cytokine responses

Splenocytes were prepared and seeded at 4 × 10^5^/well using ten wells of a 96-well plate. Recombinant PrtA2 was added at 10 μg/ml and incubated for 48 h. Then, the supernatants were pooled and stored at − 80 °C until use. The cytokine levels were measured using a DuoSet® ELISA (enzyme-linked immunosorbent assay) Development System (R&D Systems).

### PrtA-specific antibody titers

PrtA-specific isotype antibody titers were measured in the sera, saliva, nasal washes, and BALF, as described previously [37, 38]. In brief, a 96-well plate was coated with 100 ng/50 μl PrtA2 in PBS and incubated overnight at 4 °C. After blocking with a 50 μl blocking buffer [0.5% bovine serum albumin (BSA), 0.05% Tween-20, and 1 mM EDTA in PBS], 50 μl of twofold serially diluted samples were added and incubated at room temperature for 2 h. Following three washes, horseradish peroxidase-labeled goat anti-mouse IgG or rat anti-mouse IgA (Southern Biotech) was added, and the plate was incubated at room temperature for 2 h. The color was developed using tetramethylbenzidine (Science Products, Inc.). The endpoint titer was expressed as the reciprocal log_2_ of the last dilution that gave an OD of 0.1 or 0.2 at 450 nm for IgG and IgA, respectively.

To analyze the titers of PrtA-specific IgG subclasses, modified mouse Ig isotyping ELISA was used. Slightly, diluted rat anti-mouse Ig isotype antibodies (1 × 10^− 3^; IgG1, IgG2a, IgG2b, and IgG3) (Affymetrix, eBioscience) were used to detect the levels of PrtA-bound IgG subclasses in 96-well plates as described earlier. HRP-labeled donkey anti-rat Ig antibodies (Jackson ImmunoResearch) were pre-adsorbed with 2% nonimmune mouse sera to minimize the cross-reaction.

### Detection of PrtA on pneumococcal surfaces using vaccinated mouse sera

A. Flow cytometry assay

Bacteria cultured from stock for less than 13 h were diluted 1:50 in THY broth and cultured until the mid-log phase. Next, 1 ml bacterial broth was centrifuged and resuspended in filtered PBS containing 1% BSA. PrtA was detected using vaccinated mouse sera (1:20 dilution) by incubating at 37 °C for 20 min. The mouse sera collected from adjuvant-treated littermates were used as controls. The pneumococcal surface-bound Ig was detected using FITC-anti-mouse IgG antibody (eBioscience) and was analyzed by flow cytometry using a FACSCalibur™. Anti-cell wall polysaccharide (CWPS) antisera (SSI Diagnostica) were diluted 1:100 to interact with D39 cells as another control. Surface-bound Ig was identified using FITC-anti-rabbit IgG antibody (BD).

B. Immunofluorescence assay

*S. pneumoniae* D39 cells were labeled with 0.5 mg/mL FITC, as described previously [39, 40], and were resuspended in PBS. The whole-cell immunofluorescence assay was performed according to the method published by Jose et al. [41] with slightly modification. The cells dried on the round coverslips were first fixed with 4% paraformaldehyde for 20 min and then washed by PBS for three times. After blocking with 3% BSA in PBS, FITC-labeled D39 cells were incubated with 1/150 diluted antisera at room temperature for 1 h. The pneumococcal surface-bound Ig was identified using DyLight™ 649-conjugated anti-mouse IgG antibody (1:150) (BioLegend) or Cy5-anti-rabbit IgG antibody (Abcam) (1:150), and then was visualized using immunofluorescence microscopy with DeltaVision™ Elite.

### Opsonophagocytosis assay

The opsonophagocytosis assay was performed according to the method published by Martinez et al. [42], by using murine RAW264.7 macrophages. An aliquot of 20 μl of bacterial suspension containing 8 × 10^6^ CFU FITC-labeled D39 cells was mixed in a 96-well round bottom plate with 1:1 serially diluted antiserum from the vaccinated mice. The plate was incubated at 37 °C for 30 min with shaking at 200 rpm. Opsonized bacterial cells were then co-cultivated with RAW264.7 macrophages in 24-well plates, that were activated with 100 nM PMA for 3 days. The opsonophagocytosis was conducted at 37 °C for 30 min with shaking at 200 rpm. The bacterial cells were then washed twice with cold PBS, and RAW264.7 macrophages were detached with 2% EDTA in PBS. Extracellular FITC was quenched with 20 μg/ml trypan blue followed by flow cytometry analysis by using FACSCalibur™.

### Bacterial challenge of immunized mice

Bacteria cultured overnight were diluted 1:50 in THY broth and cultured until the mid-log phase. Following washing with PBS, and the OD at 580 nm was adjusted to 3.0 before aliquots were stored at − 80 °C. The bacterial counts were estimated after plating. Mice with complete vaccinations were challenged with *S. pneumoniae* D39 on day 14 after the last boost. The mice were injected with 2.5 × 10^4^ CFU of *S. pneumoniae* D39 in 100 μl PBS via the retro-orbital venous sinus or anesthetized and infected with 1–3 × 10^7^ CFU of D39 in 40 μl PBS intranasally. Bacterial counts in the blood were monitored at 1–3 days after infection. Blood was collected by submandibular bleeding using a lancet. At 1–3 days after infection, the mice were sacrificed to remove the lungs, which were homogenized to examine bacterial load during acute infection. The humane endpoint was applied to the survival experiment to reduce distress to the animals. We monitored the health of the mice every 12 h for 6 days after the bacterial challenge. If the mice were found to experience labored breathing, lethargy, or inability to ambulate, then they were killed instead of letting them suffer and progress to the experimental endpoint.

### Statistics

Significant differences between groups were analyzed using Student’s *t*-test or Mann-Whitney test. The value indicated in the figures represents mean and standard deviation (SD). The survival rate was investigated using the Kaplan–Meier estimation, and the significance was evaluated by the log-rank test.

## Results

### Expression and purification of the conserved amino terminal of PrtA

**Fig. 1:**
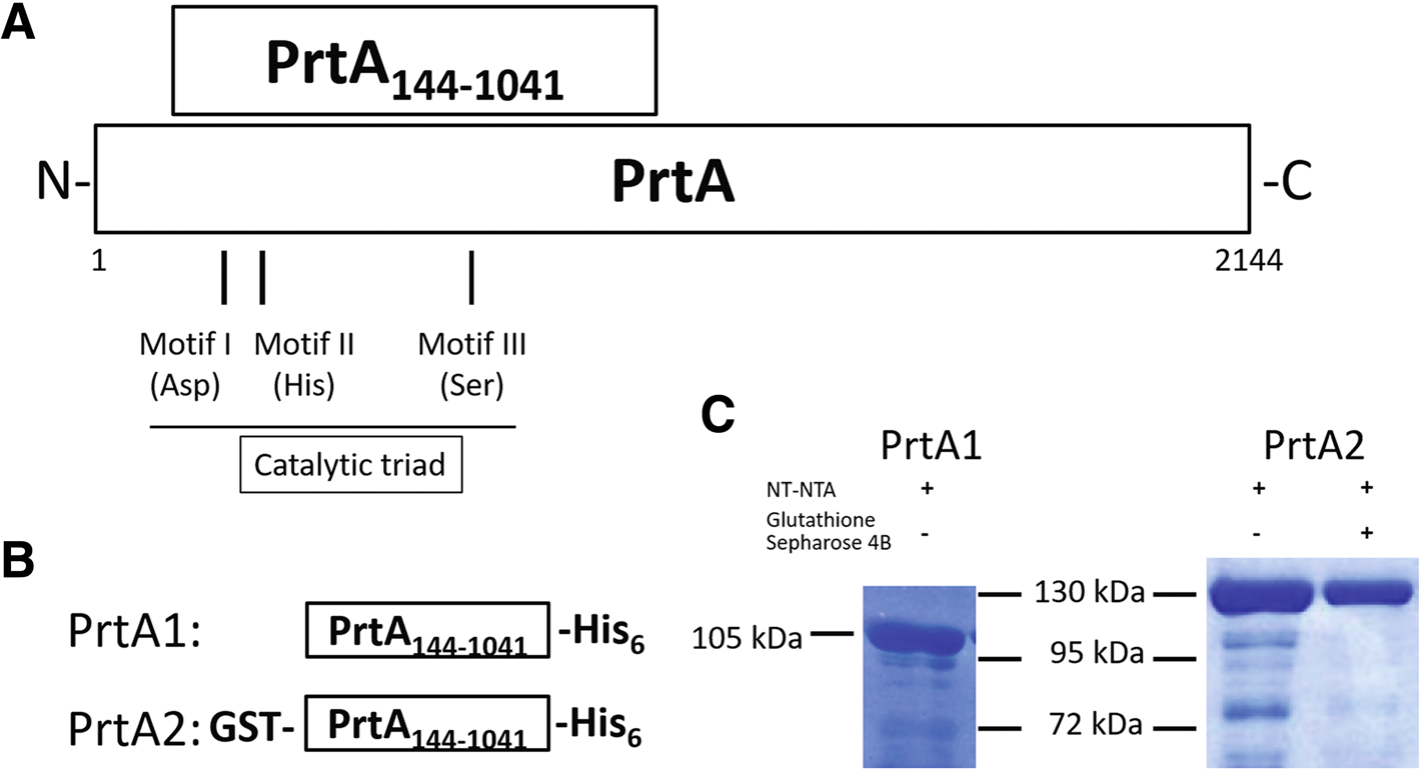
Purification of the amino terminal of PrtA. **a** The PrtA fragment (amino acids 144–1041) containing the catalytic triad was cloned and expressed. **b** Illustration of the PrtA fragment used for expression and purification. **c** PrtA1 and PrtA2 purified using Ni-NTA resin alone or with glutathione-Sepharose 4B were resolved by SDS-polyacrylamide gel electrophoresis and visualized by Coomassie Brilliant Blue staining

The PrtA fragment containing the catalytic triad was cloned into the pET29a expression vector, and its expression was induced under an optimal IPTG concentration and temperature. The expressed PrtA1 recombinant protein was in-frame with the histidine (His) tag and purified using Ni-NTA resin. Additionally, a PrtA fragment in-frame with both the GST and His tags (PrtA2) was purified using Ni-NTA resin and glutathione-Sepharose 4B. The molecular weight of PrtA1 and PrtA2 were approximately 105 and 130 kDa, respectively (Fig. 1). PrtA2 had a higher purity than PrtA1. However, PrtA2 yield dramatically decreased after complete purification. Thus, PrtA1 was used for vaccination and PrtA2 was used to analyze the antigen-specific immune response in vitro.

### PrtA1 vaccination elicited an antigen-specific cellular immune response

IL-17A is crucial in the pulmonary defense of a host, therefore, the PrtA1 immunogen was combined with curdlan to induce a strong IL-17A response when administered intranasally [43]. Since pulmonary pneumococcal infection still threatens neonates and infants in a clinical setting, immunization was induced in infant mice (3–4 weeks old) to evaluate the efficacy of vaccination. After exposure to the immunogen on three occasions, the immune response was determined 1 week after the last immunization.

**Fig. 2:**
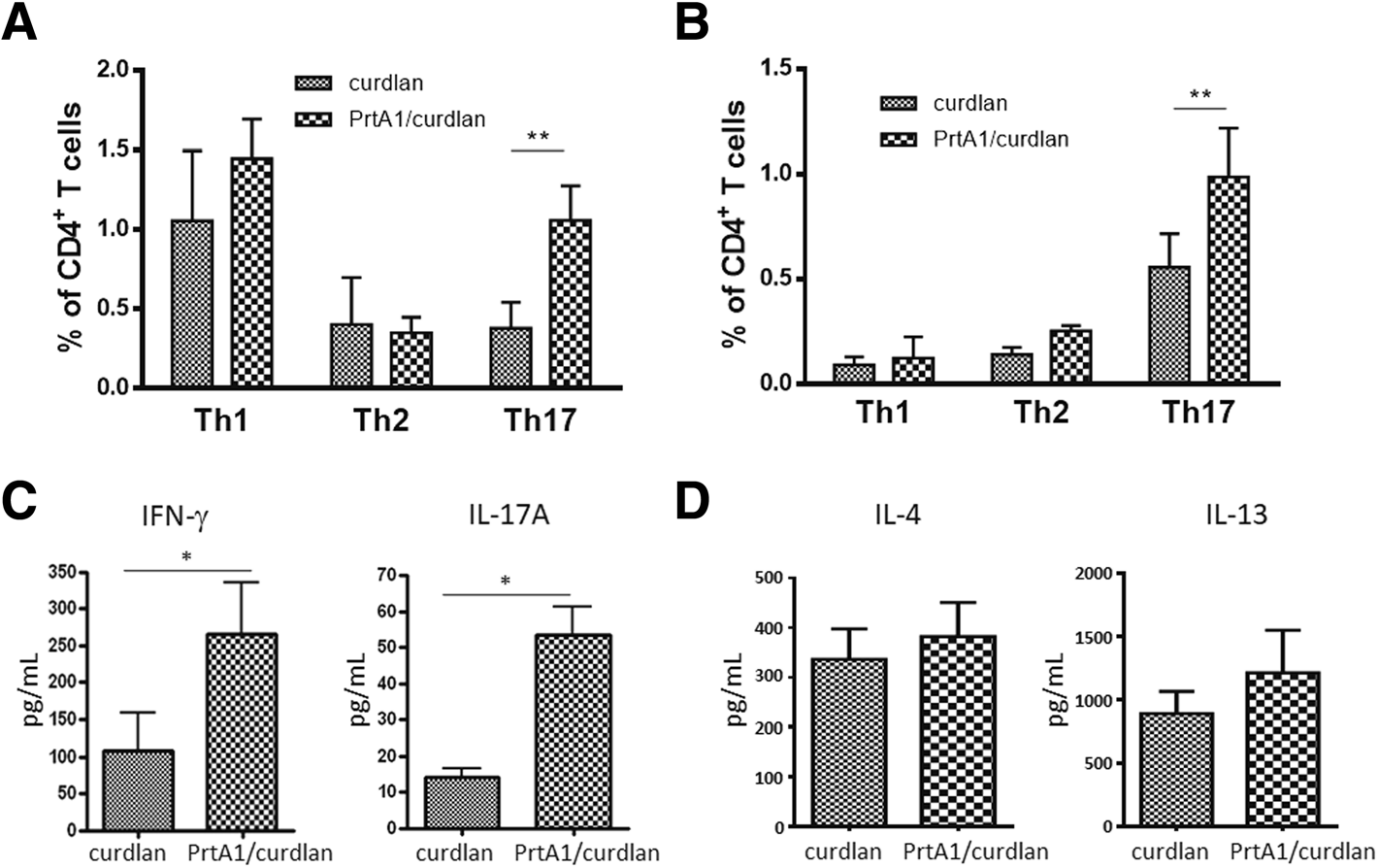
PrtA immunization induced effector T cell responses. At 3 weeks after immunization, mouse splenocytes were stimulated with (**a**) PMA and ionomycin for 6 h or (**b**) PrtA for 48 h before T cell subsets were examined by flow cytometry. The Th1 (IFN-γ^+^CD4^+^), Th2 (IL-4^+^CD4^+^), and Th17 (IL-17A^+^CD4^+^) ratios relative to the total CD4^+^ T cells were compared between the PrtA1/curdlan groups and the curdlan control group (*n* ≥ 4). **p < 0.01, Student’s *t*-test. **c** IL-17A and IFN-γ production levels after PrtA stimulation in vitro were compared between the immunization groups and the adjuvant control group (*n* = 4). *p < 0.05, Mann–Whitney test. **d** IL-4 and IL-13 production levels were detected after anti-CD3 (0.1 μg/mL) and anti-CD28 (0.2 μg/mL) antibody activation for 24 h (*n* = 5)

After three rounds of immunization, vaccine-sensitized splenocytes were treated with PMA and ionomycin to determine whether immunization with PrtA1 combined with curdlan (PrtA1/curdlan) could activate effector T cells. PrtA1/curdlan did not increase the proportion of Th1 nor Th2 cells within the total number of CD4 T cells. However, PrtA1/curdlan induced an increase in the number of Th17 cells by over twofold compared with the adjuvant control group (Fig. 2a). Antigen-recall assay showed that the spleens belonging to the mice in the PrtA1/curdlan group exhibited higher levels of antigen-specific Th17 cells than those of the mice in the adjuvant control group (Fig. 2b). After recombinant PrtA protein stimulation in vitro for 48 h, the splenocytes belonging to the PrtA1/curdlan immunization group showed a higher potential to produce antigen-specific IL-17A than those belonging to the adjuvant control group (Fig. 2c). Although in vitro stimulation of PrtA did not recall a high percentage of IFN-γ-positive CD4^+^ effector T cells (Fig. 2b), it triggered PrtA1/curdlan-immunized splenocytes to produce high levels of antigen-specific IFN-γ (Fig. 2c). In contrast, PrtA1/curdlan immunization did not induce antigen-specific Th2 cells (Fig. 2b). For the reason that the levels of PrtA-specific IL-4/IL-13 production by splenocytes were lower than the assay’s limit of detection, we measured IL-4/IL-13 levels after the splenocytes were treated with anti-CD3/CD28 antibodies for 24 h. The levels of both IL-4 and IL-13 produced by the splenocytes of mice belonging to the PrtA1/curdlan-immunized and adjuvant control groups were similar (Fig. 2d). Splenocytes from the mice immunized with PrtA1 alone produced undetectable levels of PrtA-responsive cytokines (data not presented). This indicates that curdlan induced PrtA to produce antigen-specific IL-17A and IFN-γ, but not Th2 response.

### PrtA induced an antigen-specific humoral response

**Fig. 3:**
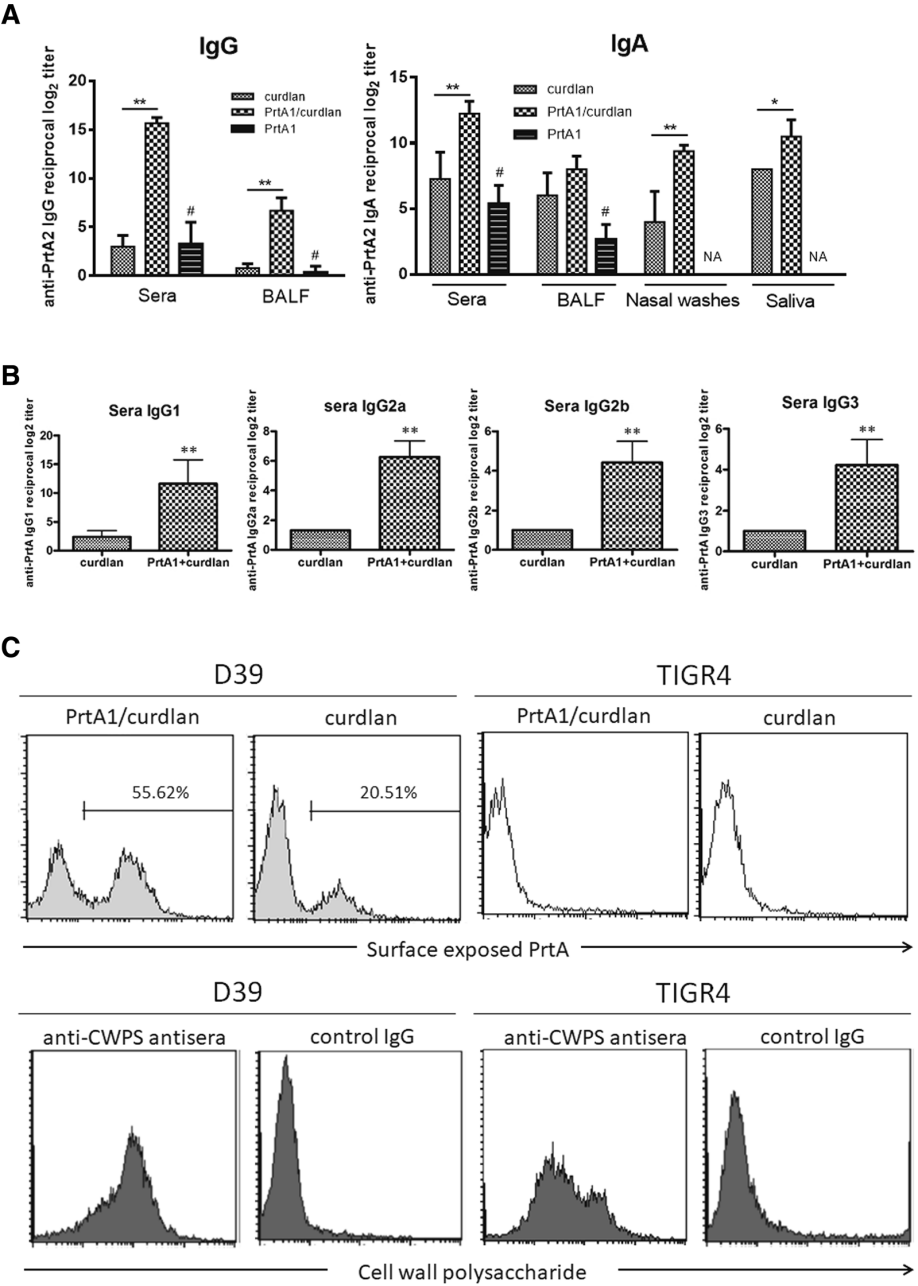
PrtA immunization increased the antigen-specific immunoglobulin titer and responses. **a** Sera, nasal washes, saliva, and the BALF were collected from the PrtA1/curdlan-immunized group and the adjuvant control group after three vaccinations (*n* ≥ 4). Sera and BALF were also collected from mice immunized with PrtA1 protein alone (*n* = 3). The antigen-specific Ig titer was measured using PrtA-specific ELISA. IgG and IgA were distinguished by an isotype-specific secondary antibody. The reciprocal log_2_ of the last dilution yielded an OD of 0.2 at 450 nm for sera IgG and 0.1 for the others. *p < 0.05; **p < 0.01; # *p* < 0.01 compared with PrtA1/curdlan, Student’s *t*-test. NA, not analyzed. **b** PrtA-specific IgG subclasses were determined by modified mouse Ig isotyping ELISA (*n* ≥ 4). **p < 0.01, Student’s *t*-test. **c** Sera from immunized mice, but not adjuvant control mice, induced pneumococcal-bound Ig in D39, but not in TIGR4. The sera were pooled from at least four mice in either immunized or adjuvant control group. Rabbit anti-CWPS antisera were used as a control. Bacterial cell-bound IgG was detected using FITC-anti-mouse IgG or FITC-anti-rabbit IgG antibodies and analyzed by flow cytometry

To assess the antibody response after PrtA immunization, sera and mucosal secretions (BALF, nasal washes, and saliva) were collected from the mice to measure PrtA-specific IgG and IgA responses. The anti-PrtA IgG titer in the sera and BALF was significantly higher after immunization with PrtA1/curdlan than that of the adjuvant control group and PrtA1-only group (Fig. 3a). This indicated that curdlan supplementation improved the PrtA-specific IgG titers in the sera and BALF. The PrtA-specific IgA titer also increased markedly in the sera when it was combined with curdlan. Mucosal secretions, saliva, and nasal washes showed higher PrtA-specific IgA titers for the immunized mice than the control mice; however, the IgA titer did not increase in BALF (Fig. 3a). The titers of the IgG subclasses after immunization were also measured and showed that PrtA-specific IgG1, IgG2a, IgG2b, and IgG3 titers of PrtA1/curdlan-immunized mice increased significantly (Fig. 3b).

To check whether the PrtA-specific antibody could bind to the surface of *S. pneumoniae* cells, *S. pneumoniae* strains D39 and TIGR4 were incubated with the sera from immunized mice and IgG binding was evaluated using an anti-mouse IgG-FITC antibody. The sera from PrtA1/curdlan-immunized mice showed that the PrtA-specific antibody bound to the surface of D39 cells [serotype 2] and not TIGR4 cells [serotype 4]; the sera from adjuvant control group failed to elicit antibodies specific to the bacterial cells (Fig. 3c). However, the anti-PrtA antisera did not interact with the entire pneumococcal population. We used an anti-cell wall polysaccharide antibody (anti-CWPS antisera, SSI Diagnostica) to rule out the possibility that the bacterial capsule impeded with the binding of the bacterial cells to the antigen-specific antibody. It showed that the anti-CWPS antisera could access the target cells independent of the capsular serotype with more than 90% of conjugation on the whole population (Fig. 3c). These results indicated that although PrtA immunization elicited antigen-specific antibodies, PrtA-immunized antisera selectively bound to the surface of the pneumococcal cells with limited potency.

### PrtA-specific antisera facilitated opsonophagocytosis of *S. pneumoniae*

**Fig. 4:**
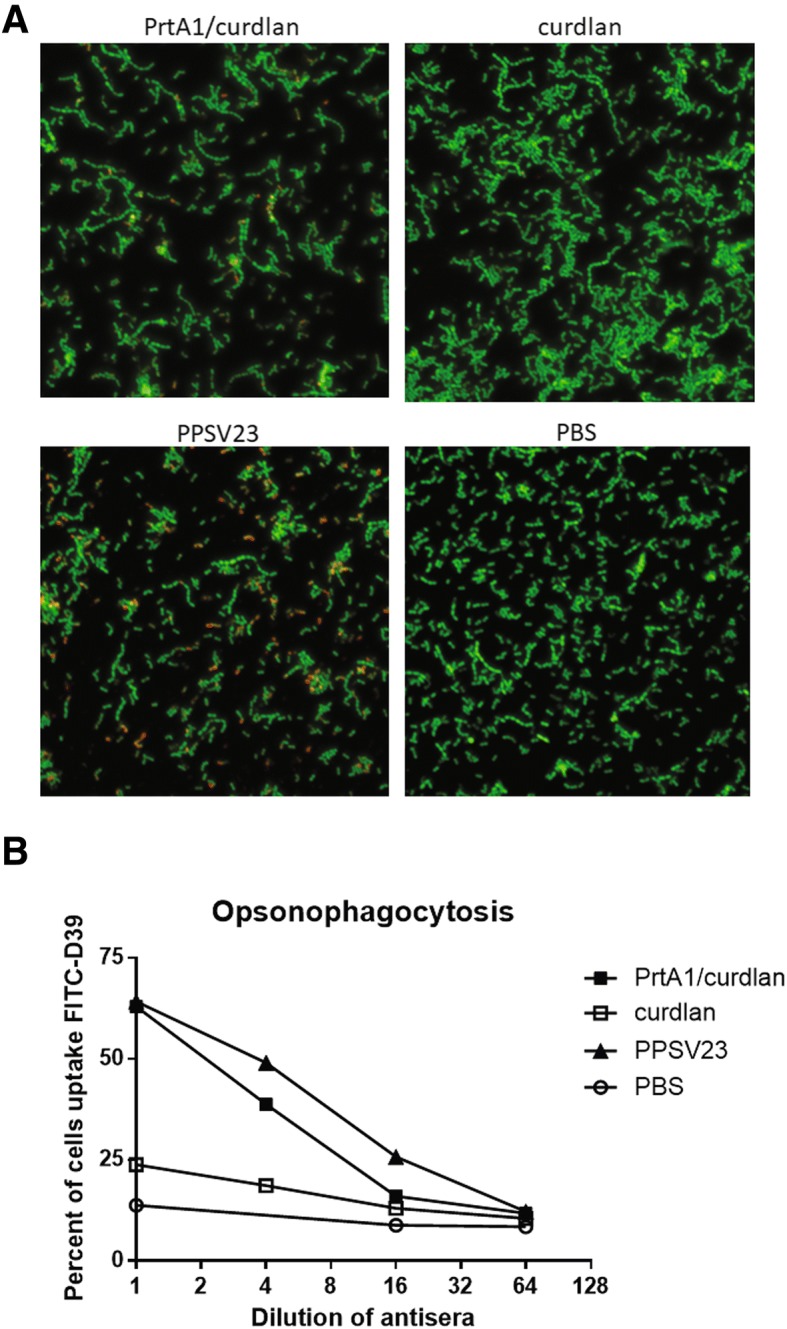
PrtA immunization facilitated opsonophagocytosis. **a** FITC-labeled *S. pneumoniae* D39 cells were incubated with pooled antisera (*n* = 4) from PrtA1/curdlan- or curdlan-immunized groups. The opsonized bacterial cells were identified using Dylight™ 649-anti-mouse IgG antibody and were visualized using immunofluorescence microscopy (DeltaVision Elite) under 40× oil objective. The images were processed by deconvolution. **b** FITC-labeled D39 cells were mixed with serially diluted antisera and were incubated with PMA-activated murine macrophage RAW264.7. The proportion of RAW264.7 cells to internalized bacterial cells was examined using flow cytometry after extracellular fluorescence quenching with trypan blue. The sera were pooled from four mice in each group, and the results were a representative of two independent experiments

We further checked the efficacy of PrtA1/curdlan-immunized antisera in assisting the opsonophagocytosis of FITC-labeled D39 cells. The capability of immunized antisera-opsonized bacterial cells was first accessed with immunofluorescence assay. It showed that PrtA-specific antisera potentiated opsonization of the D39 cells, while adjuvant control antisera bound to the D39 cell surface with little efficacy (Fig. 4a). The PPSV23-immunized antisera were used as positive control and PBS-treated control antisera functioned as parallel negative control. Compared with PPSV23, antisera from PrtA-immunized mice revealed considerably weaker activity when bound to pneumococcal surfaces, suggesting that surface-bound Ig levels were lower in PrtA-antisera than in PPSV23-antisera. Although PrtA-immunized antisera could conjugate to D39 cell surface, the distribution of surface-bound Ig signal was not well correlated with that of FITC-labeled D39 cells (Additional file 1). In contrast, signals from capsule targeting Ig in PPSV23 antisera were well distributed and superimposed on where FITC signals (D39 cells) presented (Additional file 1). It suggested portion of bacterial cells eluding PrtA-specific Ig recognition, which was in accordance with the result of our flow cytometry assay (Fig. 3c).

We then co-cultivated the activated RAW264.7 macrophages and the opsonized bacterial cells with shaking to reduce non-specific interaction. It revealed that PrtA-immunized antisera facilitated the phagocytosis of D39 cells by the macrophages (Fig. 4b) owing to its higher capability of opsonizing pneumococci compared with adjuvant control antisera. We found that the bacterial cells opsonized by PrtA-antisera could be ingested by the phagocytes as potently as by PPSV23-antisera regardless of the quantity of Ig on the surface of the bacterial cells (Fig. 4a, b). This suggests phagocytes could clear opsonized bacterial cells with similar potency irrespective of the degree of Ig conjugation on the bacterial cell surface. The data indicates that intranasal immunization with PrtA1/curdlan triggered systemic humoral immune responses and facilitated opsonophagocytosis of *S. pneumoniae* cells*.*

### PrtA combined with curdlan immunization failed to protect against pneumococcal pneumonia and systemic invasion

**Fig. 5:**
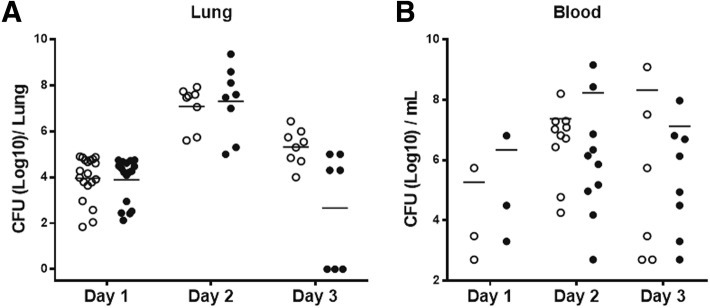
PrtA immunization failed to protect mice against acute pneumococcal pneumonia. *S. pneumoniae* D39 was intranasally administered to immunized group and the adjuvant control group. **a** Bacterial load in the lungs and (**b**) subsequent bloodstream invasion were examined 1, 2, and 3 days post infection. The open circle represents the curdlan-treated mice, and the filled circle represents the PrtA1/curdlan-immunized mice. The data were pooled from one or two experiments. At least three mice per experiment were used

PrtA1/curdlan immunization induced high IgA titers in sera as well as nasal washes and also induced a higher level of PrtA-specific IL-17A production. Therefore, we tested the efficacy of PrtA immunization against pneumococcal pneumonia. BALB/c mice with complete immunizations were challenged with 1–3 × 10^7^ CFU of *S. pneumoniae* D39 in 40 μl PBS via nasal administration. After 1–3 days of infection, bacterial colonization was examined by plating out the lung homogenates. Although PrtA immunization elicited a high IgG titer in the BALF, it did not promote D39 clearance in the lungs during acute infection (Fig. 5a). The bacterial load in the blood was further examined, and there was no significant difference in the D39 count between the immunization group and adjuvant control group (Fig. 5b). PrtA1/curdlan seemed to reduce the bacterial load in the infected lungs of the mice on day 3 and tended to alleviate the mucosal invasion; however, day 3 data might reveal a biased trend since some mice in both groups (four in curdlan group; three in PrtA1/curdlan group) died due to severe pneumonia or bacteremia at midnight, which were excluded in the experimental results. These data suggest that PrtA1/curdlan did not effectively protect the mice against *S. pneumoniae*-induced acute pneumonia and the resulting systemic invasion.

### PrtA immunization failed to suppress blood propagation of *S. pneumoniae* cells

**Fig. 6:**
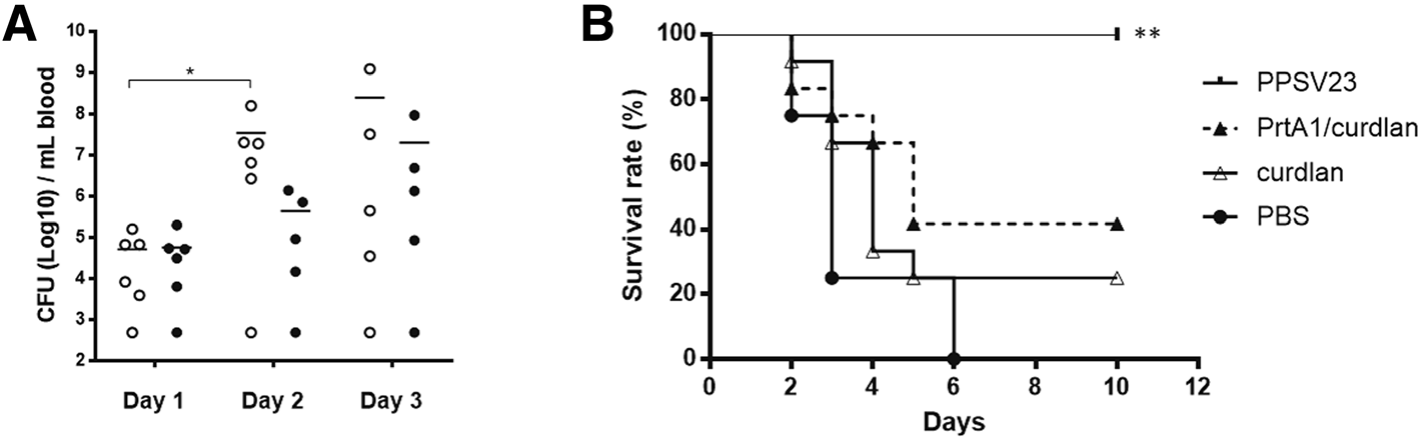
PrtA immunization failed to protect mice against blood propagation of *S. pneumoniae*. *S. pneumoniae* D39 cells were intravenously administered to the immunized group or adjuvant control group (*n* = 6 in each group). **a** The bacterial load in the blood was monitored on days 1, 2, and 3 post-infection. *n* = 6; *p < 0.05; Mann–Whitney test. **b** The survival rate was monitored after pneumococcus infection. Mice administered with PPSV23 were used as the positive control. **p < 0.01; Log-rank test

Although PrtA1/curdlan immunization increased the PrtA-specific IgA titer in most mucosal secretions as shown earlier, the rising antibody titer in paired samples seemed to be insufficient in controlling bacterial burden in the respiratory tract. Because PrtA/curdlan immunization markedly increased antigen-specific IgG and IgA titers in the sera, we checked the protective efficacy against blood propagation of *S. pneumoniae*. D39 cells were injected intravenously into immunized and nonimmunized mice, and the bacterial load after infection as well as the approximative survival rate was ascertained to evaluate the vaccine efficacy. The bacterial burden in the blood of curdlan control mice increased from day 1 to day 2, whereas it was restrained in PrtA1/curdlan immunized mice (Fig. 6a), suggesting that the PrtA-specific Ig potentiated bacterial clearance during the early infection phase. However, PrtA immunization did not increase the survival rate of mice compared with adjuvant control group or untreated mice (Fig. 6b). Clinical PPSV23 vaccine was administered as the positive control, which protected the mice from lethality. PPSV23 prevented bacterial propagation via the blood from day 1 of infection, which was examined by the plating count (data not presented). Our results indicate that although PrtA1/curdlan immunization reduced bacterial load in the blood during the early phase of infection, it failed to alleviate the severity of pneumococcal bacteremia.

## Discussion

Overall results indicate that on one hand PrtA/curdlan immunization activated antigen-specific antibody and IL-17A response in the mice, but on the other, it failed to protect against pneumococcal pneumonia and invasive infection. PrtA mediates human apolactoferrin cleavage to yield an N-terminal lactoferricin-like peptide that is more efficient than apolactoferrin as a bactericide against *S. pneumoniae* [44]. It suggested that human innate immunity provided defense against pneumococci with assistance from PrtA and considered PrtA inadequate as a vaccine candidate. It is unknown if mouse apolactoferrin is the PrtA substrate, but a *prtA* mutation reduced the virulence of *S. pneumoniae* in a murine pneumonia model [21, 22, 45], thereby suggesting that it plays a pathogenic role.

PrtA was selected using convalescent-phase serum [20] and was considered an immunogen. Zysk et al. could not confirm the immunoreactivity of PrtA based on 45 serum samples from patients with invasive pneumococcal diseases [46]. However, they used part of the *prtA* gene (nucleotides 330–1041) to express recombinant PrtA as an antigen and that it was highly hydrophobic indicated by TopPred 2 [47]; thus it might not have been an ideal T or B cell receptor ligand in all patients. Another study also observed no significant difference in the anti-PrtA IgG titer in sera from acute and convalescent patients with pneumococcal bacteremia [48]. They further indicated that anti-PrtA IgG titers were higher among healthy children with pneumococcal carriage [49], thereby suggesting that higher antibody titers are insufficient against pneumococcal colonization. In the present study, immunization with recombinant PrtA generated anti-PrtA IgG that bound to the surface of D39 pneumococci but failed to mediate protection against pneumococcal pneumonia and blood stream invasion. It is well known that prior exposure to whole *S. pneumoniae* provides immunity against pneumococcal colonization, which depends on CD4^+^ T cells and IL-17A, a neutrophil-activating cytokine [26, 50]. This suggests that the IL-17A response aids vaccine efficacy. Curdlan is an agonist of dectin-1, which activates Syk-CARD9 signaling in dendritic cells to induce Th17 differentiation [32]. As mentioned earlier, we saw that PrtA-specific IL-17A response was induced in the mice with curdlan as an adjuvant, but it did not protect against pneumococcal pneumonia. Curdlan has been previously shown to facilitate a conserved protein vaccine, PopB, in preventing *P. aeruginosa*-induced pneumonia [33]. With the potency of Th17 stimulation, it is noteworthy to mention that PopB/curdlan could induce a 30-fold higher antigen-specific IL-17A response than PrtA/curdlan, as seen in the antigen-recall T cell activation assay. The distinct IL-17A responses induced by the vaccines used in this study, combined with curdlan, might be attributable to the innate properties of the immunogens along with the potency of being conjugated with curdlan. Curdlan retains antigen in the polymer microspheres via protein-polysaccharide interaction and is extensively used as an adjuvant. High retention of immunogens with curdlan-based support would be expected to provide ideal adjuvant activity. A high molecular weight tetanus anatoxin (150 kDa) has been previously shown to provide about 16-fold lower retention on curdlan than a low molecular weight lysozyme (14.6 kDa) [51]. PrtA is 105 kDa in size which is self-evidently higher than the 40 kDa PopB, that the inefficiency of the IL-17A response induced by PrtA was probably attributable to the low retention on curdlan.

Although PopB/curdlan confers higher protection against pulmonary infection, 40% lethality is still observed within 5 days [33], which might be correlated with an antibody response that is ineffective suppressing bacterial propagation. *S. pneumoniae* D39 is a highly invasive, encapsulated strain with the ability to mediate antiphagocytosis [52]. Clinical PPSV23 vaccine offered 100% protection to mice infected with D39 due to optimal antibody binding, which results in complement deposition or opsonophagocytosis leading to effective pneumococcal clearance. Examination of the protective efficacy of PrtA immunization by determination of the bacterial load in the blood after intravenous infection revealed that the PrtA1/curdlan immunized group, but not the adjuvant control group, restrained bacteria proliferation during the first 2 days (Fig. 6a). However, it failed to protect against lethal bacteremia (Fig. 6b). We determined the potency of PrtA-antisera bound to pneumococcal surfaces and found only a portion of the pneumococcal population was conjugated (Fig. 3c, Additional file 1), which might be the point of vulnerability leading to immune escape and disease progression.

PrtA1/curdlan did not induce marked antigen-specific Th2 response, which was possibly attributable to Th1 skewing by curdlan modulation. Because Th1/2 imbalance could limit the antibody responses, we verified the efficacy of an alternative immunization with PrtA combined with traditional complete Freund’s adjuvant-incomplete Freund’s adjuvant (CFA-IFA), which was assumed to efficiently enhance global immune responses. When combined with CFA-IFA, PrtA immunization induced a considerably higher antigen-specific Th1 and Th2 response than did PrtA1/curdlan; Th17 induction was comparable with PrtA1/curdlan (Fig. 2, Additional file 2). Although CFA-IFA facilitated Th2 activation, PrtA-specific antibody titers did not improve in the sera or mucosal secretions compared with PrtA1/curdlan (Fig. 3, Additional file 3). Unlike the limited interaction of PrtA1/curdlan antisera with TIGR4 cells (Fig. 3c), the antisera from PrtA/CFA-IFA-immunized mice could successfully recognize D39 as well as TIGR4 cells (Additional file 3), possibly because of the T-cell receptor repertoire induced by CFA-IFA [53]. However, the potency of antigen-specific antibody conjugation on the pneumococcal cell surface was still limited (Additional file 3). Moreover, it failed to improve bacterial clearance from the blood and could not stop the progression of bacteremia (Additional file 4).

The number of effective immunoglobulins from both PrtA-immunized antisera (PrtA/curdlan and PrtA/CFA-IFA) bound to pneumococcal cell surfaces was lower than that from PPSV23-immunized antisera (Additional file 1). This suggested that fewer epitopes on the pneumococcal cell surface were recognized by the PrtA-antisera than by PPSV23 antisera. Moreover, higher number of pneumococci were not conjugated by Ig in PrtA-antisera than in PPSV23 antisera, highlighting the imperfect reactivity of PrtA-antisera against the pneumococci due to population heterogeneity [54, 55]. The inefficiency of PrtA/curdlan antisera was not different from that of PPSV23 antisera in the opsonophagocytosis assay (Fig. 4b). It seemed that the ratio of bacterial cells opsonized by either PrtA- or PPSV23 antisera versus phagocytes in the assay could be large enough to ignore the proportion of bacterial cells eluding Ig recognition. These escaped pneumococci might have continued to replicate; thereby, breaching the infectivity threshold. This is in accordance with the outcome of the systemic invasion murine model, which restrained bacteria load in day 2 but could not stop the bacterial propagation and disease severity to the end of experiment. The mechanism underlying the variable expression of PrtA on the pneumococcal surface remains unclear, and if elucidated, might partly explain the failure of PrtA immunization against *S. pneumoniae* invasion.

Although the PrtA/curdlan vaccine did not suppress pneumococcal invasion, we found that it tended to reduce the bacterial load in the lung after 3 days of infection, in addition to seemingly restricting the bacterial propagation during the early phase of blood invasion, despite the limited potency of the antibody response. We have previously found that BABL/c mice might not be suitable for evaluating vaccine efficacy because they are more resistant to *S. pneumoniae* infection than other mouse strains [56]. Furthermore, a high dose of infection, which is needed to potentiate pneumococcal disease, could exceed the threshold of protection conferred by the PrtA/curdlan vaccine. This limitation might also partly explain the failure of the PrtA vaccine.

Global efforts are being made to develop efficient protein vaccine candidates against *S. pneumoniae*. An effective mucosal Th2 response and IgA titer, in addition to antigen-specific Th17 are essential to prevent pneumococcal colonization of the lungs. A high titer of antigen-specific IgG and sufficient potency to bind to the surface of all bacteria in population, are critical to prevent pneumococcal invasion. Thus, the variable expression of selected pneumococcal vaccine candidates needs to be considered in the field.

## Conclusions

Combining a PrtA fragment (amino acids 144–1041) with curdlan induced antigen-specific Th17 and antibody response in BALB/c mice after immunization. However, the PrtA/curdlan vaccine did not effectively protect the mice against acute pneumonia, along with failing to suppress pneumococcal propagation in the blood. The failure of the vaccine might be attributable to the limited potency of PrtA-specific antibodies bound to the pneumococcal cell surface.

## Additional files

Additional file 1: PrtA-induced antibody conjugated with pneumococci with limited potency. FITC-labeled *S. pneumoniae* D39 cells were incubated with antisera as indicated. The opsonized bacterial cells were identified using Dylight™ 649-conjugated secondary antibody and were visualized by immunofluorescence microscopy (DeltaVision Elite) under 40× oil objective. The inserts show higher magnification (with 60× oil objective) of Ig-conjugated bacteria. The images were processed by deconvolution. The antisera were pooled from at least four mice in either immunized or adjuvant control group. (TIF 19843 kb)

Additional file 2: PrtA1/CFA-IFA immunization induced effector T cell responses. PrtA1 combined with 100 μL of CFA or IFA (PrtA/CFA-IFA) was injected subcutaneously in the neck of the mice with the same antigen dosage and administration schedule as the curdlan adjuvant (see details in Materials and Methods). PrtA1, CFA, and IFA were mixed sufficiently to create an emulsion before immunization with the CFA mixture for the first week and the IFA mixture for the following two weeks. One week after completion of the immunization, the spleens were isolated from immunized mice. (A) The splenocytes were stimulated with PMA and ionomycin for 6 h before T cell subset examination by flow cytometry. Th1 (IFN-γ^+^CD4^+^), Th2 (IL-4^+^CD4^+^), and Th17 (IL-17A^+^CD4^+^) numbers relative to the total CD4^+^ T cells were compared between the immunized groups and the adjuvant control group (*n* ≥ 4). (B) After recombinant PrtA protein stimulation in vitro for 48 h, the supernatant was collected for cytokine analysis. *p < 0.05; **p < 0.01; Student’s *t*-test. (TIF 11486 kb)

Additional file 3: PrtA1/CFA-IFA immunization increased the antigen-specific immunoglobulin titer and responses. (A) Sera, BALF, and nasal washes were collected from the immunized mice and the adjuvant control group (n ≥ 4) one week after three vaccinations. The antigen-specific Ig titer was measured using PrtA-specific ELISA. IgG and IgA were distinguished using an isotype-specific secondary antibody. The reciprocal log_2_ of the last dilution yielded an OD of 0.2 at 450 nm for sera IgG and 0.1 for the others. **p < 0.01; Student’s *t*-test. (B) Pneumococcal-bound IgG in antisera from vaccinated mice and adjuvant control mice were evaluated using two pneumococcal strains, D39 and TIGR4. Bacterial-bound IgG was detected using an FITC-anti-mouse IgG antibody and flow cytometry. The ratio was representative of the percentage of total pneumococci being conjugated by antigen-specific IgG. (C) FITC-labeled *S. pneumoniae* D39 cells were incubated with antisera from PrtA1/CFA-IFA-immunized or adjuvant control groups. The opsonized bacterial cells were identified using Dylight™ 649-anti-mouse IgG antibody and were visualized by immunofluorescence microscopy (DeltaVision Elite) under 40× oil objective. The images were processed by deconvolution. The antisera used in (B) and (C) were pooled from at least four mice in either immunized or adjuvant control groups. (TIF 9038 kb)

Additional file 4: PrtA1/CFA-IFA immunization failed to protect mice against pneumococcal bacteremia. *S. pneumoniae* D39 cells were intravenously administered to immunized group or adjuvant control group (*n* ≥ 6). (A) The bacterial loads in the blood from PrtA1/CFA-IFA- or CFA-IFA- immunized mice were monitored on days 1, 2, and 3 post infection. *p < 0.05; Mann–Whitney test. (B) The survival rate was monitored after pneumococcal infection. The humane endpoint was replaced with experimental endpoint. (TIF 13629 kb)

## Abbreviations

BALF: Bronchoalveolar lavage fluid
CFA: Complete Freund’s adjuvants
ELISA: Enzyme-linked immunosorbent assay
IFA: Incomplete Freund’s adjuvants
IFN: Interferon
IL: Interleukin
Th: T helper
